# GABA Receptor Activation in Müller Glia as a Molecular Switch for Controlling VEGF-A in the Retina

**DOI:** 10.1080/17590914.2026.2618997

**Published:** 2026-02-01

**Authors:** Alan E. Medina-Arellano, Jesus Silvestre Albert-Garay, Karla Tovar-Hernandez, Matilde Ruiz-Cruz, Lenin Ochoa-de la Paz

**Affiliations:** a Laboratorio de Neurobiología Molecular y Celular de la Glía, Departamento de Bioquímica, Facultad de Medicina, Universidad Nacional Autónoma de México (UNAM), Coyoacán, Ciudad de México, México; b Unidad de Investigación, Asociación Para Evitar la Ceguera en México I.A.P, Ciudad de México, México; c Programa de Doctorado en Ciencias Biomédicas, UNAM, México

**Keywords:** Calcium, GABA receptors, Müller cells, retina, VEGF

## Abstract

GABA receptors are classically known for driving neuronal hyperpolarization and modulating synaptic transmission. In glial cells, however, GABA induces depolarization and triggers calcium-dependent signaling pathways. Müller glia, the principal retinal glial population, maintain retinal homeostasis and are the major source of neuroretinal VEGF-A, a key angiogenic factor in development and disease. Although GABA receptor (GABAR) activity has been proposed to influence retinal VEGF-A, it remains unclear whether this regulation occurs through Müller glial cells (MGC) and which mechanisms are involved. Here, we investigated how GABAR activation modulates VEGF-A in primary mouse MGC cultures. Cells were exposed to GABA and selective agonists or antagonists of GABA_A_ (muscimol, gabazine) and GABA_B_ receptors (baclofen, CGP55845). VEGF-A expression and secretion were analyzed by immunofluorescence, western blot, RT-qPCR, and ELISA. To assess Ca^2+^ involvement, we used Ca^2+^-free Ringer-Krebs solution and the L-type channel blocker nimodipine, and examined MAPK signaling with the ERK1/2 inhibitor FR180204. Our findings show that GABA and muscimol increased VEGF-A fluorescence intensity after 48 hours while reducing VEGF-A secretion, without altering *Vegfa* mRNA. Both effects were abolished by extracellular Ca^2+^ removal or nimodipine, indicating a Ca^2+^-dependent mechanism. FR180204 also attenuated GABA- and GABA_A_-mediated effects, implicating MAPK signaling. Short-term assays revealed that GABA rapidly elevates VEGF-A protein and secretion within ∼30 minutes. Together, these findings identify a Ca^2+^- and GABA_A_-dependent pathway through which Müller glia regulate VEGF-A production and release, providing new insight into glial signaling and neurotransmitter-driven modulation of retinal angiogenic factors.

## Introduction

Müller glial cells (MGCs) are the most abundant glial cells in the mammalian retina and perform several essential functions to maintain homeostasis, including K^+^ buffering, neurotransmitter recycling, and growth factor secretion (Reichenbach & Bringmann, 2013). MGCs express a wide range of neurotransmitter receptors, such as glutamatergic, glycinergic, serotonergic, and GABAergic receptors. GABA signals through membrane receptors called GABA_A_ (GABA_A_R) and GABA_B_ (GABA_B_R), and both types have been observed in MGCs across multiple mammalian species, including humans (Biedermann et al., 2002, 2004; Hinds et al., 2013; Reichelt et al., 1996, 1997). In MGCs, GABA_A_ receptors help regulate Cl^-^ and pH balance (Biedermann et al., 2004), and some research suggests they also modulate intracellular Ca^2+^ dynamics (Hinds et al., 2013). Interestingly, in glial cells unlike neurons, activating GABA_A_ receptors causes depolarization, which then opens voltage-gated Ca^2+^ channels (VGCC) and increases intracellular Ca^2+^ levels (Gilbert et al., 1984; Káradóttir & Attwell, 2007; Kettenmann et al., 1984, 1987; Kettenmann & Schachner, 1985; Plotkin et al., 1997).

Several studies show that GABA receptors (GABAR) play a role in maintaining retinal blood vessel stability. For example, missing the GABA_A_R rho-1 subunit in genetically modified mice leads to increased retinal blood vessel leakage, along with higher levels of pro-angiogenic genes like VEGF-A (Vascular Endothelial Growth Factor A) and IGF-1 (Insulin-like Growth Factor 1), and lower levels of anti-angiogenic genes such as PEDF (Pigment Epithelium-Derived Factor), compared to control animals (Zheng et al., 2010). Additionally, intravitreal injection of midazolam, a benzodiazepine, prevents retinal vascular leakage and neovascularization in diabetic mice (Lee et al., 2022). These studies indicate that retinal GABA_A_Rs may help maintain vascular stability, although the specific cellular players and mechanisms involved are still unknown.

Since MGC are known to be the main source of VEGF-A release in the neuroretina (Saint-Geniez et al., 2008; Wang et al., 2010), this study aimed to explore whether MGC can influence the synthesis and release of VEGF-A in response to GABA receptor activation. We present a new mechanism involving activation of the ionotropic GABA_A_R, subsequent activation of the L-type Ca^2+^ channel (LTCC), and extracellular Ca^2+^ influx, ultimately leading to activation of the ERK/MAPK signaling pathway. We suggest that MGC may serve as an important cellular component that regulates VEGF-A secretion following GABA_A_R activation.

## Materials and Methods

### Animals and Müller Cell Primary Cultures

Experiments were approved by the Comité Interno para el Cuidado y Uso de Animales de Laboratorio (CICUAL) at the Facultad de Medicina, Universidad Nacional Autónoma de México (#018-CIC-2019). Primary Müller cell cultures were obtained from CD-1 mice (P4-7). Briefly, the mice were euthanized by decapitation, and both eyes were enucleated. Eyecups were incubated overnight at 4 °C with gentle agitation in DMEM/F12 supplemented with 10% fetal bovine serum (FBS) (S1620, Biowest, USA) and 1% penicillin/streptomycin (15140122, Gibco, USA) (DMEM-suppl). The following day, the retinas were dissected, mechanically dissociated in DMEM-suppl, and seeded in 60 mm Petri dishes containing DMEM-suppl plus human epidermal growth factor (hEGF). Cultures were maintained at 37 °C in 5% CO_2_ and passaged at 90% confluence. All experiments were conducted with cells at passage 3.

### Experimental Treatments and Pharmacology

All reagents were sourced from Sigma-Aldrich (USA): GABA (A2129), muscimol (M1523), gabazine (S106), baclofen (G014), CGP55845 hydrochloride (CGP55, SML0594), BAPTA-AM (A1076), nimodipine (N149), and FR180204 (SML0320). Stock solutions were prepared in water and diluted into 2% FBS DMEM/F12 medium to reach final concentrations: GABA at 12.5, 25, 50, 100, and 200 µM; gabazine at 10 µM; baclofen at 100 µM; CGP55845 at 5 µM; nimodipine at 10 µM; and FR180204 at 10 µM.

Ca^2+^-free Ringer-Krebs solution was prepared with the following concentrations (mM): 120 NaCl, 5 KCl, 1.3 MgCl_2_, 1.2 NaH_2_PO_4_, 26 NaHCO_3_, 5.5 D-glucose, and 10 HEPES, pH 7.4.

### Inmunofluorescence

MGC (8 × 10^3^ cells per well) were seeded on 48-well plates and treated with various experimental conditions or controls for 48 hours. After treatment, cells were washed once with PBS 1x, fixed in 4% paraformaldehyde for 20 minutes, and permeabilized/blocked with PBS containing 0.2% Triton X-100 and 10 mg/ml BSA for 1 hour. Cells were then incubated overnight at 4 °C with primary antibodies against CRALBP (1:100, sc-59487, Santa Cruz Biotechnology, USA), Sox-2 (1:100, sc-365964, Santa Cruz Biotechnology, USA), Nestin (1:250, ab221660, Abcam, USA), GABA_A_R .1-6 subunits (1:100, sc-376282, Santa Cruz Biotechnology, USA), GABA_B_R B1 subunit (1:100, sc-398901, Santa Cruz Biotechnology, USA), VEGF-A (1:250, sc-7269, Santa Cruz Biotech, USA) and HIF-1α (1:500, 36169, Cell Signaling). The following day, cells were incubated with Alexa Fluor 647-conjugated goat anti-mouse IgG (1:500) (A21235, Thermo Fisher, USA) for 1 h at room temperature. Cell nuclei were counterstained with DAPI (1:1000) (D1306, Thermo Fisher, USA) for 15 minutes at room temperature. Immunofluorescence imaging was performed using a Cytation 5 multi-imaging system (BioTek, USA). Five random fields per condition were captured at 10× magnification. Fluorescence intensity per cell was quantified with Gen 5.0 software. Data are presented as mean fluorescence intensity ± SEM, or as a fluorescence intensity histogram, which was generated by measuring the fluorescence intensity of individual cells and grouping them into bins to assess changes in VEGF-A expression at the single-cell level.

### ELISA Assays

MGC (8 × 10^3^ cells/well) were seeded in 48-well plates, and a 48-hour treatment was applied. Conditioned media were centrifuged at 420 × g for 5 minutes and stored at −20 °C. VEGF-A release was measured using a mouse VEGF-A ELISA kit (#RAB509, Sigma-Aldrich, USA) according to the manufacturer’s instructions. VEGF-A release levels were calculated from the standard curve (*r*^2^ ≥ 0.99).

### Western Blotting

MGCs were seeded in 60 mm Petri dishes until they reached confluence. Cells were exposed to various experimental treatments multiple times, then washed twice with 1× PBS, scraped, and centrifuged at 420 × g for 5 minutes. Cell pellets were lysed using RIPA buffer (R0278, Sigma-Aldrich, USA) supplemented with protease and phosphatase inhibitors. Lysates were frozen at −80 °C, thawed, sonicated, and centrifuged at 7,500 × g for 30 minutes at 4 °C. Protein concentration was measured using a BCA assay. Equal amounts (25 μg) of samples were denatured in Laemmli buffer for 5 minutes at 100 °C, separated on 15% SDS-PAGE gels, and transferred to PVDF membranes. Membranes were blocked with TBS-Tween 20 0.1% containing 5% skim milk for 1 hour at room temperature, then incubated overnight at 4 °C with primary antibodies against VEGF-A (1:100, sc-7269, Santa Cruz Biotechnology, USA) and β-tubulin (1:500, sc-5274, Santa Cruz Biotechnology, USA) in TBS-Tween 20 (0.1%) with 1% BSA. Subsequently, membranes were incubated with HRP-conjugated goat anti-mouse IgG (1:8000, Thermo Fisher, USA). Bands were visualized via chemiluminescence, quantified by densitometry using ImageJ (NIH, USA), and normalized to β-tubulin. Data were expressed as optical density arbitrary units.

### RT-qPCR

MGCs were seeded in 60 mm Petri dishes until reaching confluence. Cultures were washed with 1× PBS and lysed in 200 µL of TRIzol (15596026, Invitrogen, USA). Total RNA was extracted using phenol-chloroform, and its purity (A_260_/A_280_ > 1.8) was verified. cDNA was synthesized from 400 ng of RNA with the High-Capacity cDNA Reverse Transcription Kit (4374967, Thermo Fisher Scientific, USA). qPCR was performed with 100 ng of cDNA per reaction on a Qiagen Rotor-Gene Q system using the QuantiNova SYBR Green PCR Kit (208054, Qiagen, USA). Primer sequences are *Vegfa* Forward: GACTTGTGTTGGGAGGAGGA, Reverse: TCTGGAAGTGAGCCAATGTG; Gapdh Forward: ACTGGCATGGCCTTCCGTGTTCCTA, Reverse: TCAGTGTAGCCCAAGATGCCCTTC. Relative expression was calculated using the ΔΔCt method, normalized to *Gapdh*.

### Statistical Analysis

Graphs show the mean ± standard error of the mean (SEM) from at least three independent experiments. Normality was assessed using the Kolmogorov-Smirnov test. Parametric data were analyzed with Student’s t-test or one-way ANOVA followed by Tukey’s post-hoc test, while non-parametric data were evaluated using the Mann-Whitney U test or the Kruskal-Wallis test. All statistical analyses were conducted in GraphPad Prism v8.0.2. A *p*-value below 0.05 was considered statistically significant.

## Results

**Figure 1.**
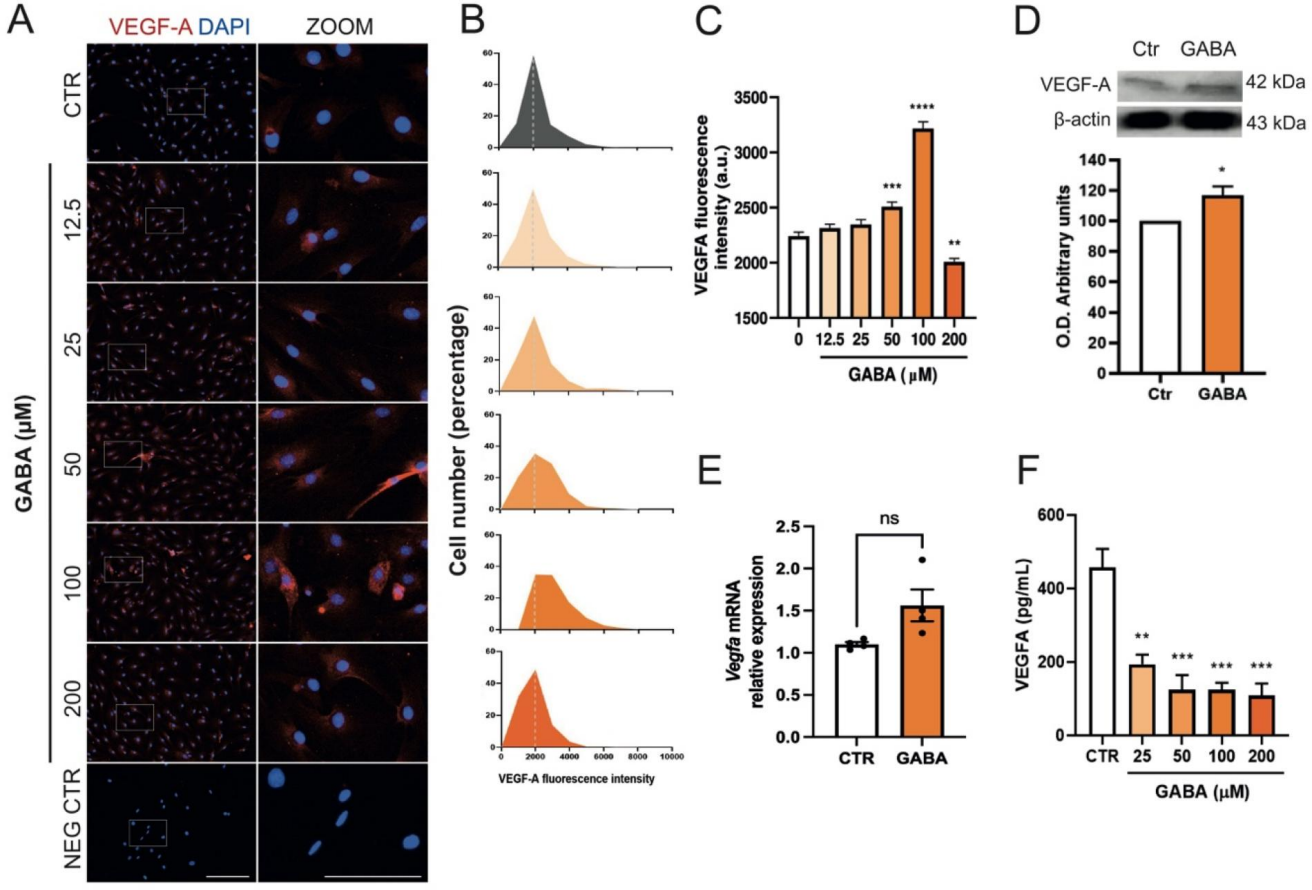
GABA regulates VEGF-A in Müller glial cell cultures. (A) Representative immunofluorescence images of MGC treated with 0, 12.5, 25, 50, 100, or 200 µM GABA for 48 hours, stained for VEGF-A (red). Nuclei were counterstained with DAPI (blue). Insets (white boxes) show 2x digital magnification. Scale bar: 200 µm (main panel) and 100 µm (insets). (B) Histogram displaying the percentage distribution of fluorescence intensity for VEGF-A to MGC cultures exposed to GABA treatments in A. (C) Average VEGF-A fluorescent intensity (mean ± SEM; *n* = 5 fields per condition). (D) Western blot and analysis of cellular VEGF-A in MGC treated with 100 µM GABA for 48 hours. E. RT-qPCR measurement of *Vegfa* mRNA levels in MGC treated with 100 µM GABA for 48 hours, normalized to *Gapdh* (*n* = 4 independent cultures). F. ELISA measurement of secreted VEGF-A in conditioned media from MGC treated with 12.5 to 200 µM GABA for 48 hours. Data were analyzed using Student’s t-test and one-way ANOVA with Tukey’s post hoc test; **p < 0.01, ***p < 0.001, ****p < 0.0001, n.s.: not significant versus control.

To determine if GABA increases VEGF-A protein expression in MGC, we performed a 48-hour dose-response assay with GABA concentrations ranging from 0 to 200 µM and measured VEGF-A levels using immunofluorescence. The analysis showed a significant rise in VEGF-A fluorescence intensity at 50 and 100 µM GABA, with no changes at lower doses. Notably, treatment with 200 µM GABA significantly decreased VEGF-A fluorescence intensity (Figure 1A,C). Fluorescence intensity values from individual cells were binned to generate population-level distributions, expressed as histograms to allow direct comparison across treatment groups. This single-cell analysis revealed a clear shift in the fluorescence distribution following GABA exposure: a subset of Müller glial cells exhibited markedly increased VEGF-A intensity, indicating that GABA selectively upregulates VEGF-A in a fraction of the population. This redistribution toward higher-intensity bins suggests underlying cellular heterogeneity in MGC responsiveness to GABA (Figure 1B). Western blotting confirmed increased intracellular VEGF-A in MGC treated with 100 µM GABA (Figure 1D), despite no changes in *Vegfa* mRNA levels (Figure 1E). Conversely, ELISA showed a significant reduction in VEGF-A secretion after GABA treatment (Figure 1F). These results suggest that GABA causes intracellular accumulation of VEGF-A protein in MGC, preventing its release.

**Figure 2.**
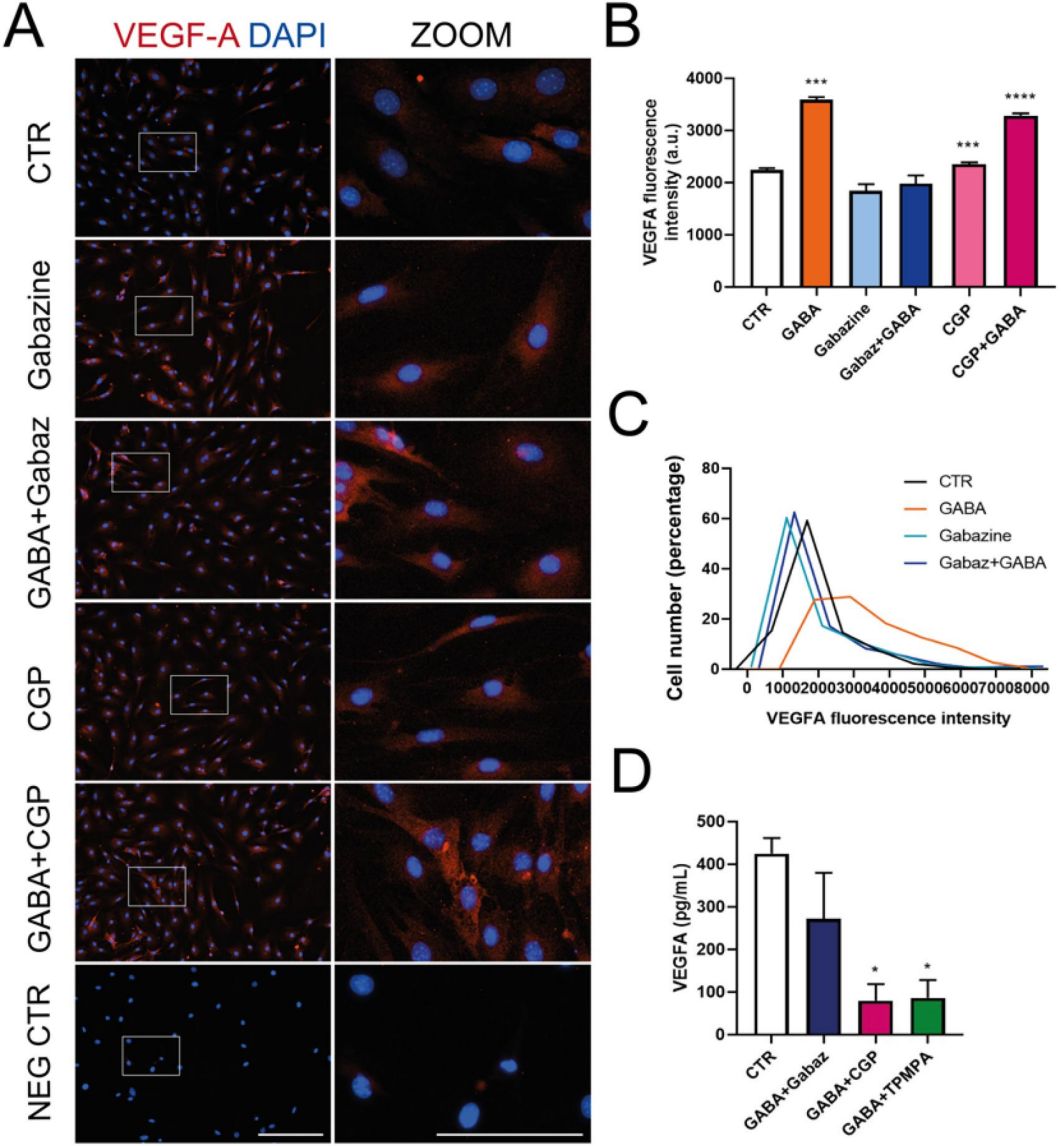
GABA_A_R blockade inhibits GABA-induced VEGF-A modulation in MGC. (A) Immunofluorescence images of MGC treated for 48 hours with 100 µM GABA alone or co-treated with 10 µM gabazine (GABA_A_R antagonist) or 5 µM CGP55845 (GABA_B_R antagonist), stained for VEGF-A (red). Nuclei were counterstained with DAPI (blue). Insets (white boxes) show 2x digital zoom. Scale bars: 200 µm (main image) and 100 µm (insets). (B) Mean VEGF-A fluorescence intensity for each condition. (C) Histogram showing the percentage distribution of VEGF-A fluorescence intensity in MGC cultures exposed to the treatments in A. (D) ELISA measurements of VEGF-A release in media from MGC treated for 48 hours with 100 µM GABA alone or co-treated with 10 µM gabazine (GABA_A_R antagonist), 5 µM CGP55845 (GABA_B_R antagonist), or 10 µM TPMPA (GABA_rho_R antagonist). One-way ANOVA with Tukey’s post hoc: *p < 0.05, **p < 0.01, ****p < 0.0001 versus control.

GABAR effects were confirmed by co-incubating GABA with a GABAR antagonist. Gabazine (10 µM), CGP55845 (5 µM), and TPMPA (10 µM) were used to block GABA_A_, GABA_B_, and GABAρ responses, respectively. The concentrations of these antagonists were chosen to replicate receptor-mediated responses previously observed in Müller glia and other glial preparations, and to ensure effective blockade under our experimental conditions (Biedermann et al., 2004; Le Meur et al., 2012; Pétriz et al., 2014; Price et al., 2008). Co-incubation of GABA with gabazine reduced GABA-induced regulation of VEGF-A, but co-application with CGP55845 did not affect the GABA response (Figure 2A,B). The histogram of VEGF-A fluorescence intensity per cell confirms that gabazine blocks the increase in VEGF-A fluorescence caused by GABA (Figure 2C). To determine if GABA_A_R effects are also influenced by GABArho receptors (GABA-R), a subclass of GABA_A_R with distinct subunits and pharmacological properties, GABA was combined with TPMPA, a selective GABA-R antagonist. Result show that VEGF-A secretion was not abolished by co-application of TPMPA (Figure 2D).

**Figure 3.**
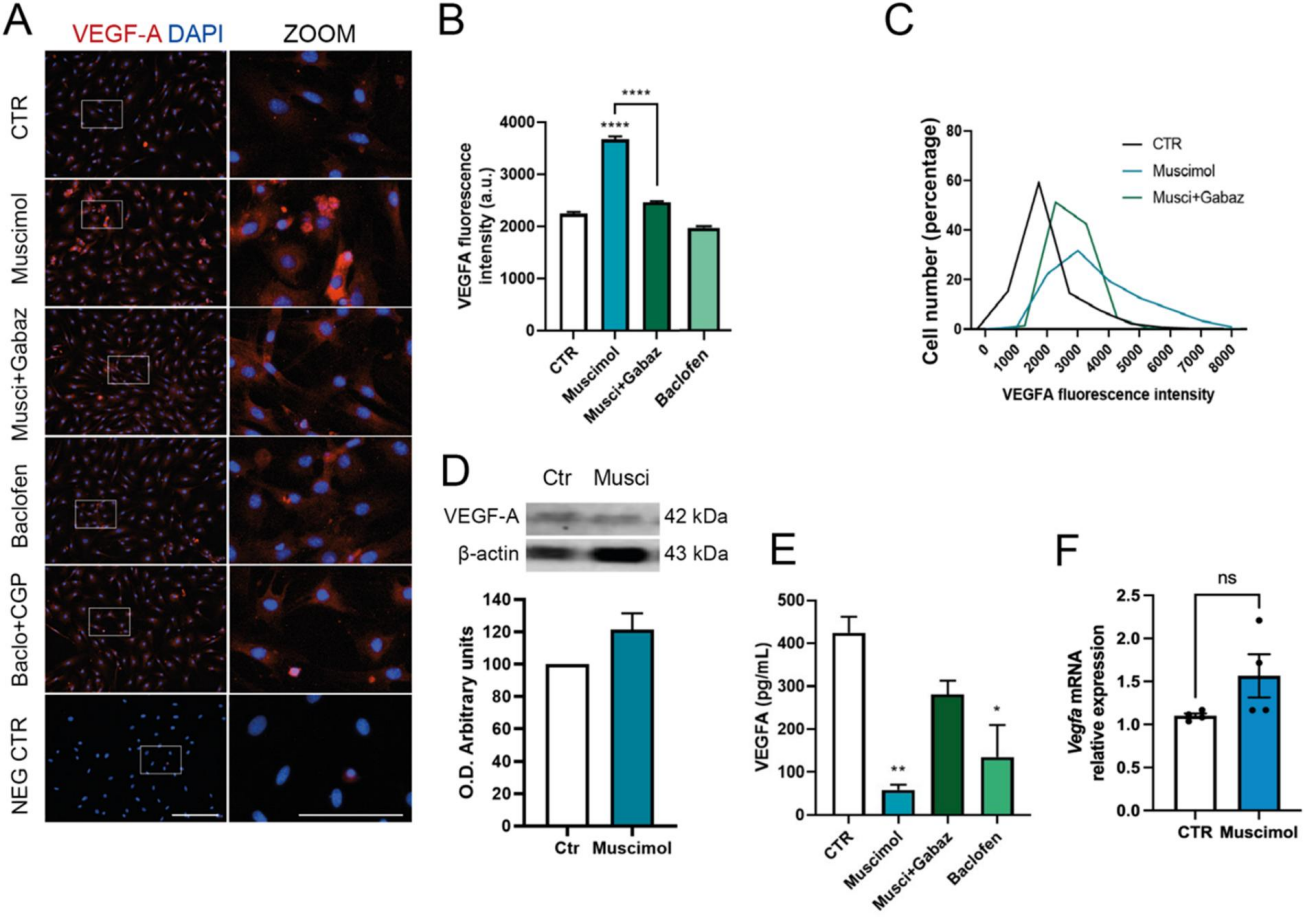
Selective GABA_A_R activation regulates VEGF-A in MGC. (A) Representative immunofluorescence images of MGC treated for 48 hours with 100 µM muscimol (GABA_A_R agonist) or 100 µM baclofen (GABA_B_R agonist), with or without gabazine at 10 µM or 5 µM CGP55845 (GABA_A_R and GABA_B_R antagonists, respectively), immunostained against VEGF-A (red). Nuclei were counterstained with DAPI (blue). Insets (white boxes) show 2× digital zoom. Scale bar: 200 µm (main panel) and 100 µm (insets). (B) Mean VEGF-A fluorescent intensity. (C) Percent distribution histogram of single-cell VEGF-A fluorescence intensities. (D) Western blot and quantification of cellular VEGF-A in MGC treated with 100 µM muscimol for 48 hours. E. Quantification of *Vegfa* mRNA in MGC treated with 100 µM muscimol for 48 hours, normalized to *Gapdh* (*n* = 4 independent cultures). F. ELISA quantification of secreted VEGF-A in conditioned media from MGC treated with 100 µM muscimol or 100 µM baclofen, with or without gabazine at 10 or 5 µM CGP55845 for 48 hours. Data were analyzed using Student’s t-test and one-way ANOVA with Tukey’s post hoc test: *p < 0.05, **p < 0.01, ***p < 0.001, ****p < 0.0001, n.s.: not significant versus control.

To determine receptor specificity, we used pharmacological agents targeting GABA_A_R and GABA_B_R. Muscimol (100 µM) was chosen based on electrophysiological studies of human Müller cells, where it elicited strong muscimol-evoked currents in this cell type (Biedermann et al., 2004). Baclofen (100 µM) served as a GABA_B_ agonist at concentrations commonly used in MGC pharmacology (Biedermann et al., 2004). Incubating MGC cultures with 100 µM muscimol, a selective GABA_A_R agonist, increased VEGF-A fluorescence intensity (Figure 3A-C). However, muscimol did not significantly change VEGF-A levels as measured by western blot (Figure 3D) or *Vegfa* mRNA levels by RT-qPCR (Figure 3F). Activating GABA_A_R specifically reduced VEGF-A secretion in MGCs (Figure 3E). The effects of muscimol were blocked by co-application of gabazine, a GABA_A_R antagonist. Interestingly, 100 µM baclofen, a GABA_B_R agonist, influenced VEGF-A release but did not alter VEGF-A fluorescence intensity (Figure 3A,B,E). These findings suggest that GABA_A_R plays a key role in the GABA-VEGF-A signaling pathway.

**Figure 4.**
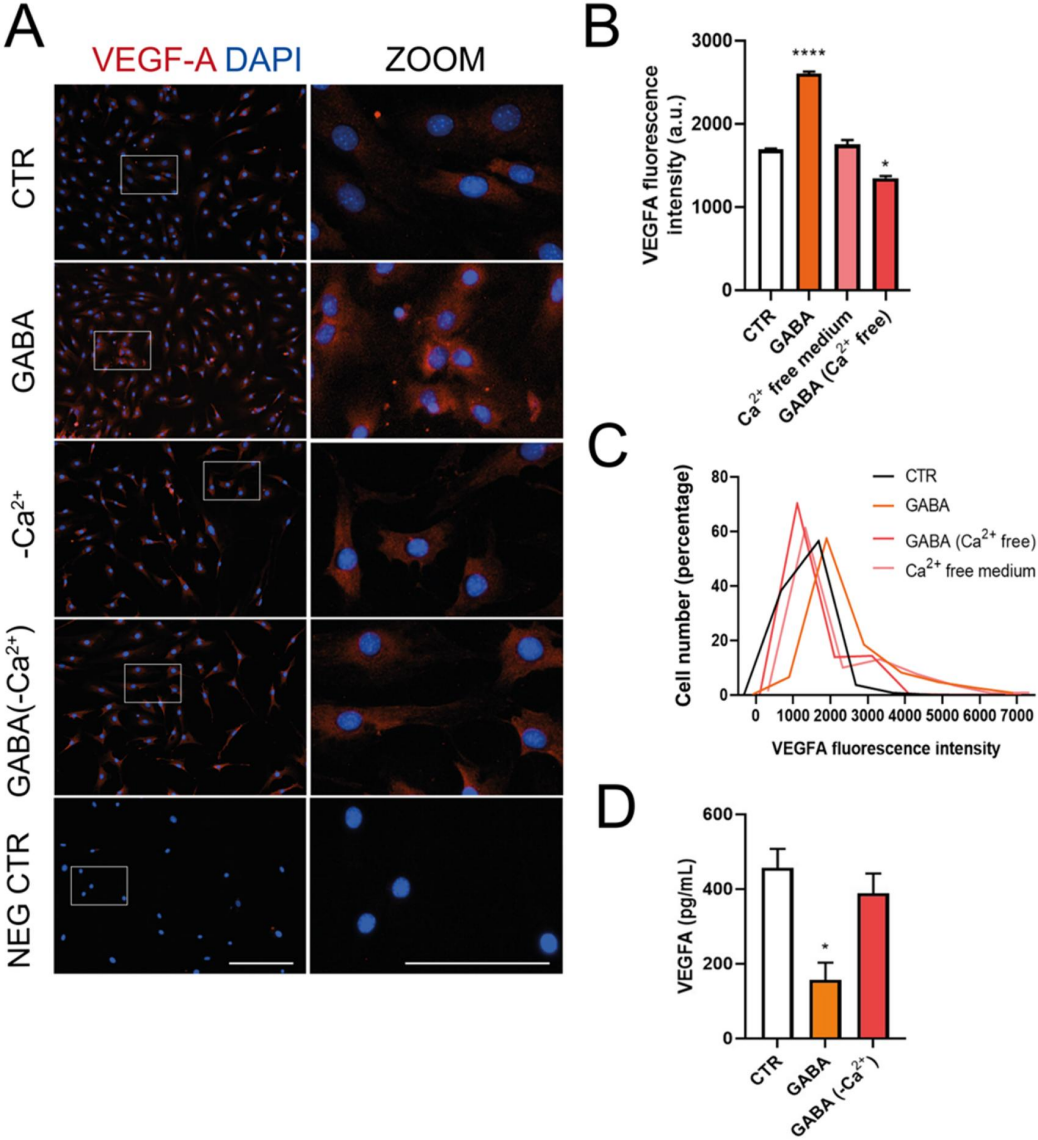
Calcium is crucial for GABA_A_R-induced VEGF-A regulation in mouse MGC. (A) Representative images showing VEGF-A expression (red) in MGC exposed to 100 µM GABA for 48 hours, with or without a Ca^2+^-free Ringer-Krebs solution (-Ca^2+^) or 10 µM nimodipine. Nuclei are counterstained with DAPI (blue). Each image includes a digital zoom (white box). Scale bars: 200 µm (original) and 100 µm (zoom). (B) Quantification of the average fluorescence intensity of VEGF-A. (C) A histogram showing the percentage distribution of VEGF-A fluorescence intensity in MGC cultures exposed to the treatments in A. (D) VEGF-A secretion by MGC after 48 hours of various treatments. ANOVA with Tukey’s test. *p < 0.05, ****p < 0.0001, compared to control.

**Figure 5.**
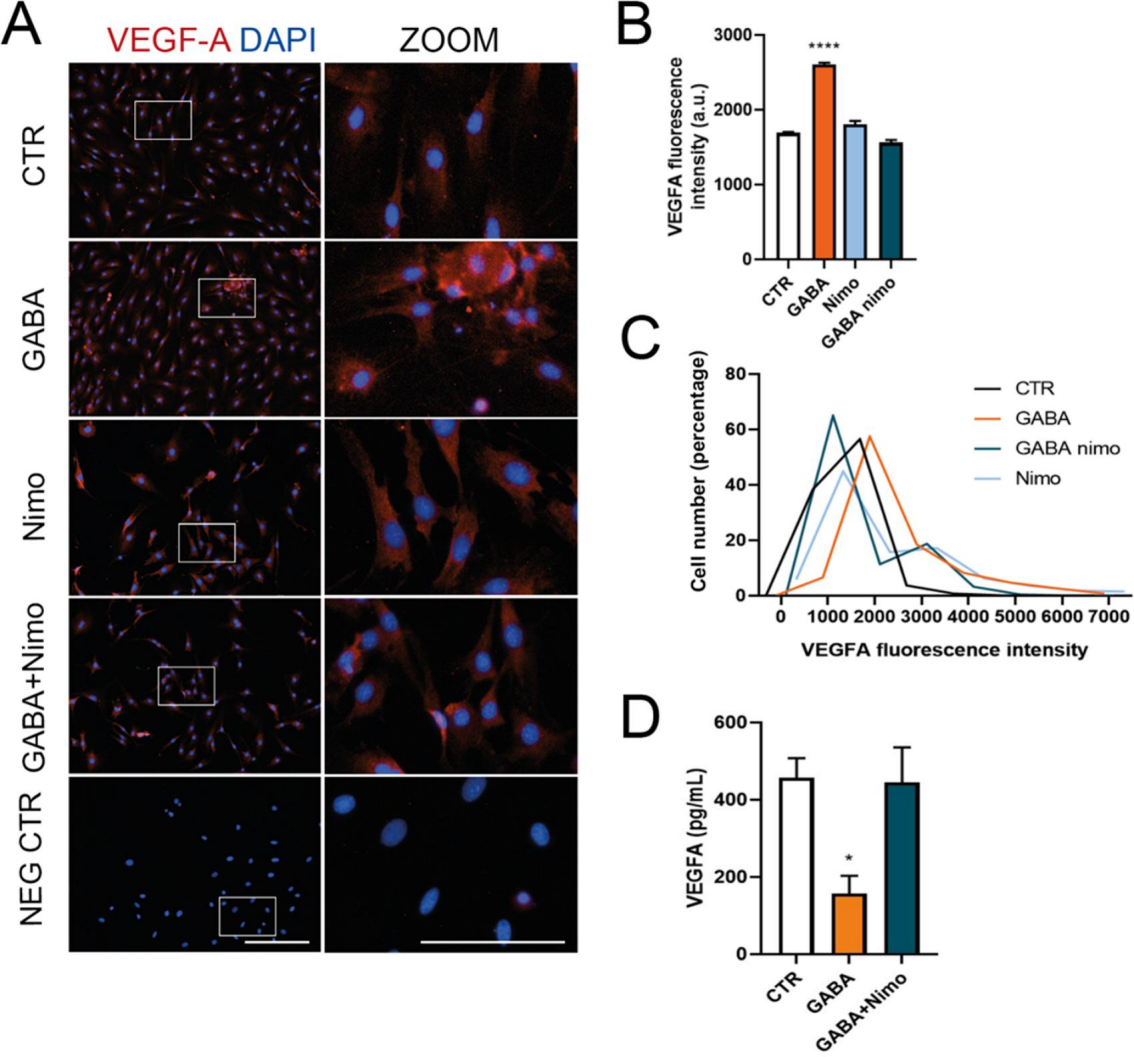
LTCC channels are involved in GABA_A_R-induced regulation of VEGF-A in mouse MGCs. (A) Representative images showing VEGF-A expression (red) in MGCs exposed to 100 µM GABA for 48 hours, with or without 10 µM nimodipine, an L-type calcium channel (LTCC) blocker. Nuclei are counterstained with DAPI (blue). Each image includes a digital zoom (white box). Scale: 200 µm (bar in original image) and 100 µm (bar in zoom). (B) Quantification of the average fluorescence intensity of VEGF-A. (C) Histogram displaying the percentage distribution of VEGF-A fluorescence intensity in MGC cultures treated as in A. (D) VEGF-A secretion levels measured in MGCs after different treatments for 48 hours. ANOVA with Tukey post hoc test. *p < 0.05, ****p < 0.0001, compared to control.

Since GABA_A_R activation depolarizes glia through Ca^2+^ influx via LTCC (Bazargani & Attwell, 2016; Gilbert et al., 1984; Kettenmann et al., 1987; Kettenmann & Schachner, 1985; Nilsson et al., 1993; Young et al., 2010), and MGC’s LTCC channels are sensitive to blockade by nimodipine (Puro et al., 1996; Yang et al., 2016), we examined the role of these channels in the GABA-VEGF-A mechanism. When MGC cultures were incubated with GABA in a Ca^2+^-free Ringer-Krebs solution, the increases in VEGF-A fluorescence intensity caused by GABA were abolished (Figure 4A–C). The absence of extracellular Ca^2+^ also restores VEGF-A secretion in the presence of GABA (Figure 4D). These data suggest that VEGF-A modulation in MGC depends on extracellular calcium. Additionally, when MGCs were incubated with 10 µM nimodipine, an LTCC blocker, the GABA-induced increases in VEGF-A fluorescence intensity were inhibited (Figure 5A–C). When VEGF-A secretion was quantified, LTCC blockade prevented GABA-induced reduction (Figure 5). These findings indicate that extracellular calcium influx through LTCC plays a role in VEGF-A regulation by GABA__A_ receptors in MGC.

**Figure 6.**
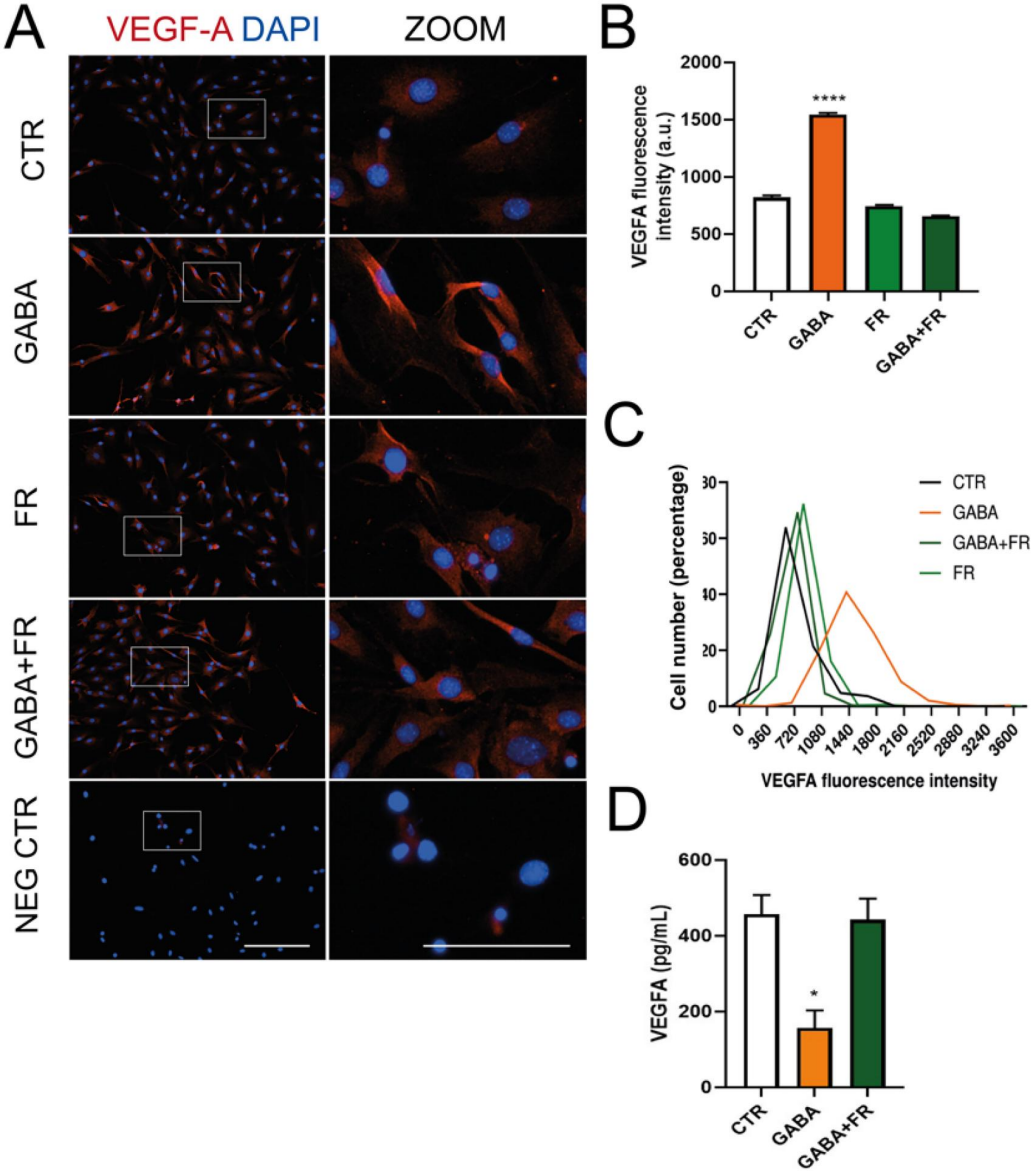
Inhibition of the MAPK signaling pathway blocks GABA_A_R-induced VEGF-A regulation in mouse MGC. (A) Representative immunofluorescence images showing VEGF-A expression (red) in MGC treated for 48 hours with 100 µM GABA, with or without 10 µM FR180204, an ERK1/2 kinase activity inhibitor. Nuclei are counterstained with DAPI (blue). Insets (white boxes) display 2× digital magnification. Scale bars: 200 µm (main panel) and 100 µm (insets). (B) Average VEGF-A fluorescent intensity (mean ± SEM; *n* = 5 fields/condition). (C) Histogram showing the percentage distribution of fluorescence intensity for VEGF-A in MGC cultures exposed to the treatments in A. (D) ELISA measurement of secreted VEGF-A in conditioned media from MGC treated with 100 µM GABA, with or without 10 µM FR180204 for 48 hours. Data were analyzed by one-way ANOVA with Tukey’s post hoc test: *p < 0.05, ****p < 0.0001.

In MGC, VEGF-A synthesis and secretion are closely connected to MAP kinase signaling (Wang et al., 2010; Ye et al., 2012). To determine whether this pathway is involved, we used FR180204, an ERK1/2 inhibitor. Incubating MGC with FR180204 in the presence of GABA prevented the increase in VEGF-A fluorescence intensity (Figure 6A-C). Additionally, inhibiting ERK1/2 with FR180204 restored VEGF-A secretion in MGC (Figure 6D). These findings suggest that the MAPK pathway plays a role in this regulatory process.

**Figure 7.**
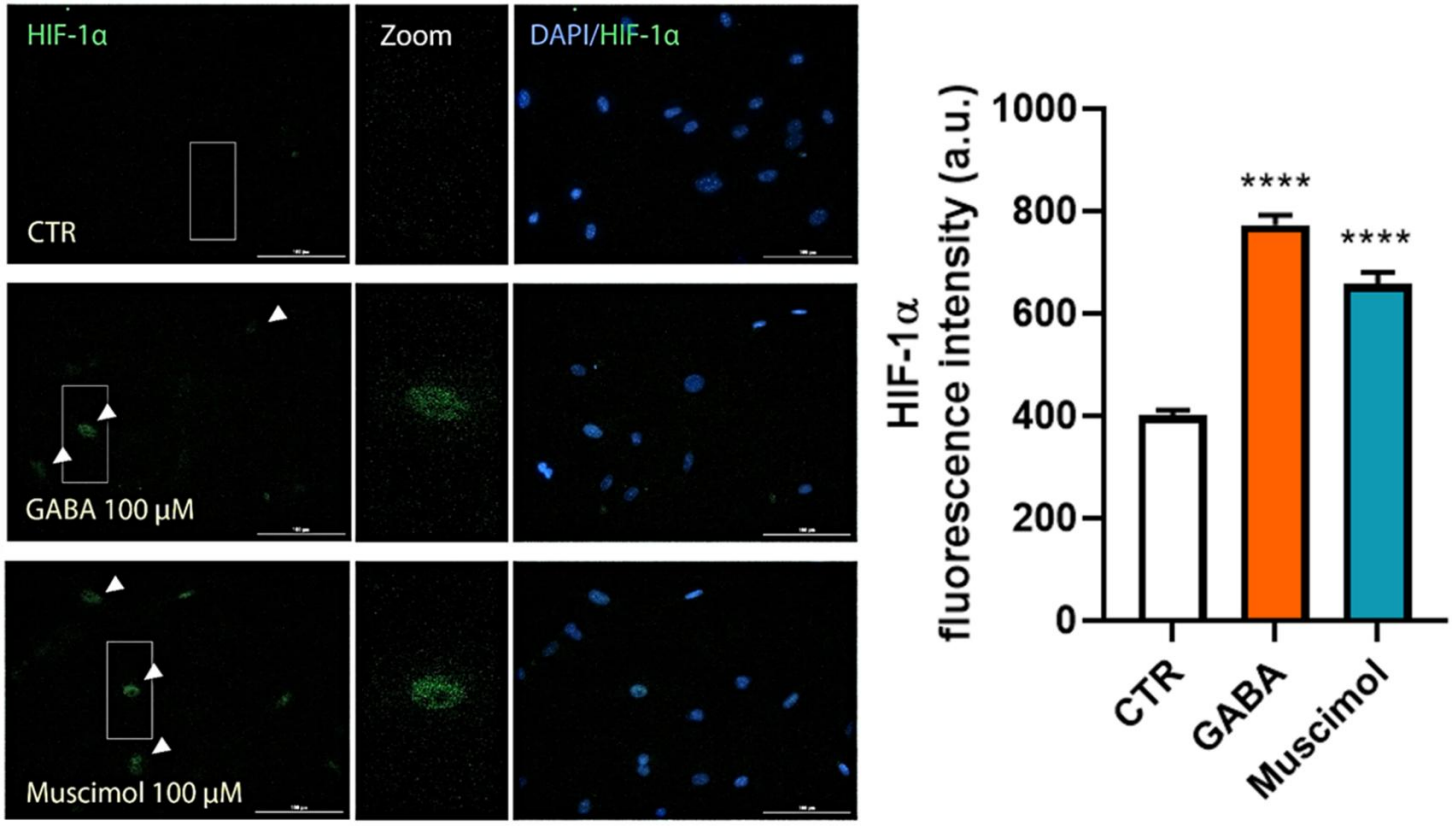
GABAergic stimulation increases nuclear HIF-1 immunoreactivity in Müller glial cells. (A) Representative immunofluorescence images of HIF-1 (green) in MGC exposed to 100 µM GABA or 100 µM muscimol for 48 hours. Nuclei were counterstained with DAPI (blue). Arrowheads indicate HIF-1-positive nuclei. Insets (white boxes) show digital magnification of selected nuclei. Scale bars: 100 µm. (B) Quantification of mean nuclear HIF-1 fluorescence intensity for each experimental condition. Data are shown as mean ± SEM from *n* = 3 independent cultures. One-way ANOVA followed by Tukey’s post hoc test, ****p < 0.0001 versus control condition.

Hypoxia-inducible factor 1α (HIF-1α) is a key transcriptional regulator that acts as a cellular oxygen sensor and promotes the expression of many pro-angiogenic genes, including VEGF-A (Lee et al., 2022; Xin et al., 2013). To investigate whether GABA_A_ receptor activation affects the nuclear accumulation of HIF-1α, we performed immunofluorescence on primary Müller glial cells treated with 100 µM GABA or 100 µM muscimol for 48 hours. Representative images show an increased nuclear HIF-1α signal in both treatment groups (Figure 7A), and quantification confirmed a significant rise compared to control (Figure 7B). These findings suggest that GABAergic stimulation correlates with increased nuclear HIF-1α immunoreactivity in Müller glia *in vitro*.

**Figure 8.**
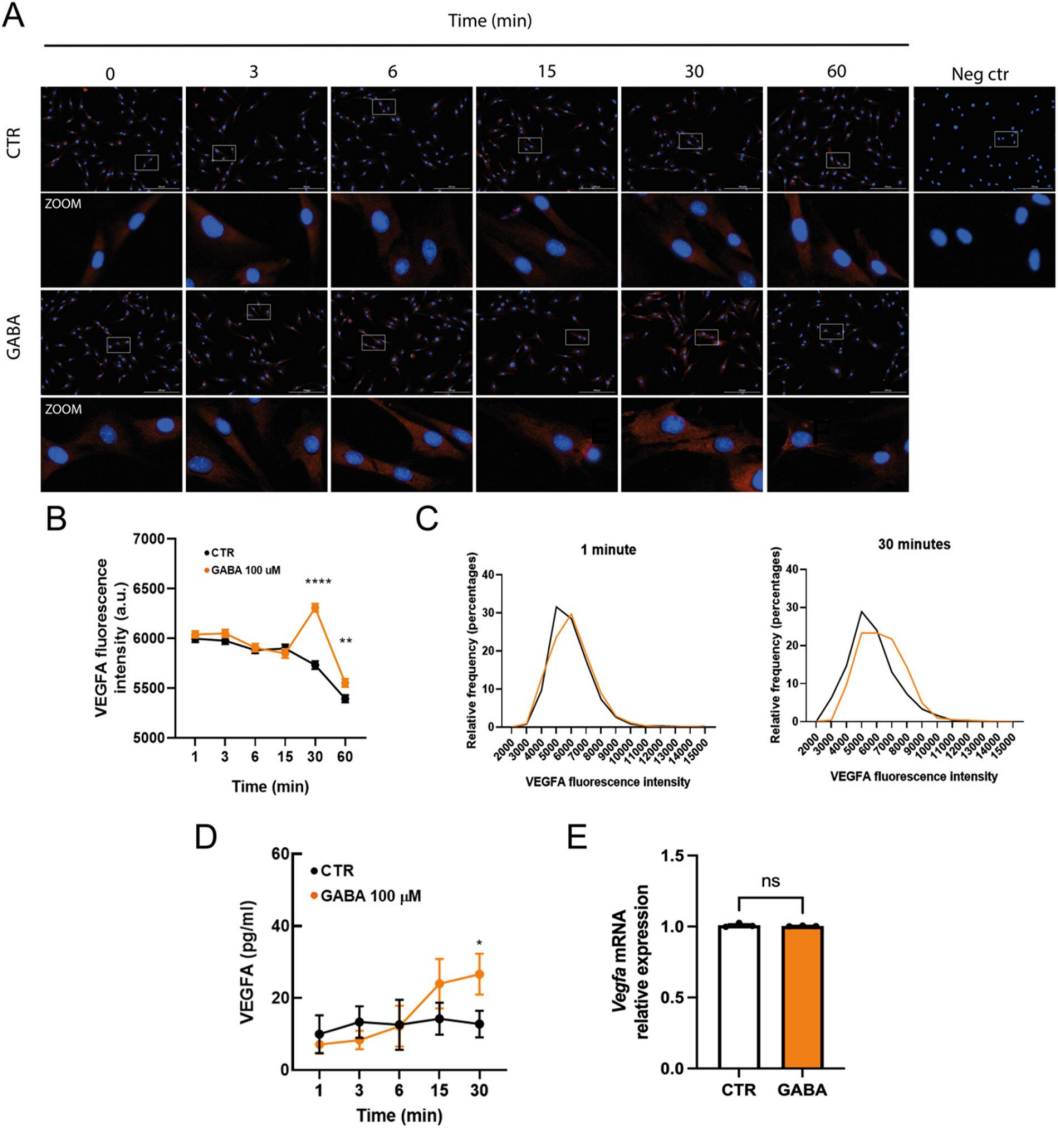
GABA induces rapid VEGF-A release in Müller glial cells. A. Time-course immunofluorescence of VEGF-A (red) in MGC exposed to 100 µM GABA for 3, 6, 15, 30, or 60 minutes. Nuclei were counterstained with DAPI (blue). Insets (white boxes) show 2x digital magnification. Scale bars: 200 µm (central panel) and 100 µm (insets). B. Average VEGF-A fluorescence intensity per field over time. C. Histogram displaying the percentage distribution of VEGF-A fluorescence intensity in MGC cultures exposed to GABA for 0 and 30 minutes. D. ELISA measurement of VEGF-A in conditioned media following 0, 3, 15, and 30 minutes of GABA exposure. E. RT-qPCR analysis of *Vegfa* mRNA at 30 minutes. Student’s t-test and one-way ANOVA with Tukey’s post hoc test: *p < 0.05, **p < 0.01, ****p < 0.0001, versus control.

Finally, we examined the short-term changes in VEGF-A regulation over time. The time-course analysis showed a significant increase in VEGF-A fluorescence intensity thirty minutes after exposing cells to 100 µM GABA, compared to the control (Figure 8A,B). When single-cell fluorescence intensities were binned and expressed as a percentage of the population, the distribution shifted to the right, indicating an increase in VEGF-A fluorescence in MGC (Figure 8C). Interestingly, an increase in VEGF-A secretion was observed at thirty minutes after 100 µM GABA exposure (Figure 8D). RT-qPCR confirmed that *Vegfa* mRNA levels did not change at 30 minutes of GABA exposure (Figure 8D). These data suggest that GABA induces rapid VEGF-A secretion, likely through LTCC-mediated Ca^2+^ signaling downstream of GABA_A_R activation, without new transcript synthesis. Overall, these findings suggest that GABA stimulates VEGF-A expression and release in mouse MGCs via GABA_A_R activation, LTCC-dependent Ca^2+^ influx, and MAPK signaling.

## Discussion

**Figure 9.**
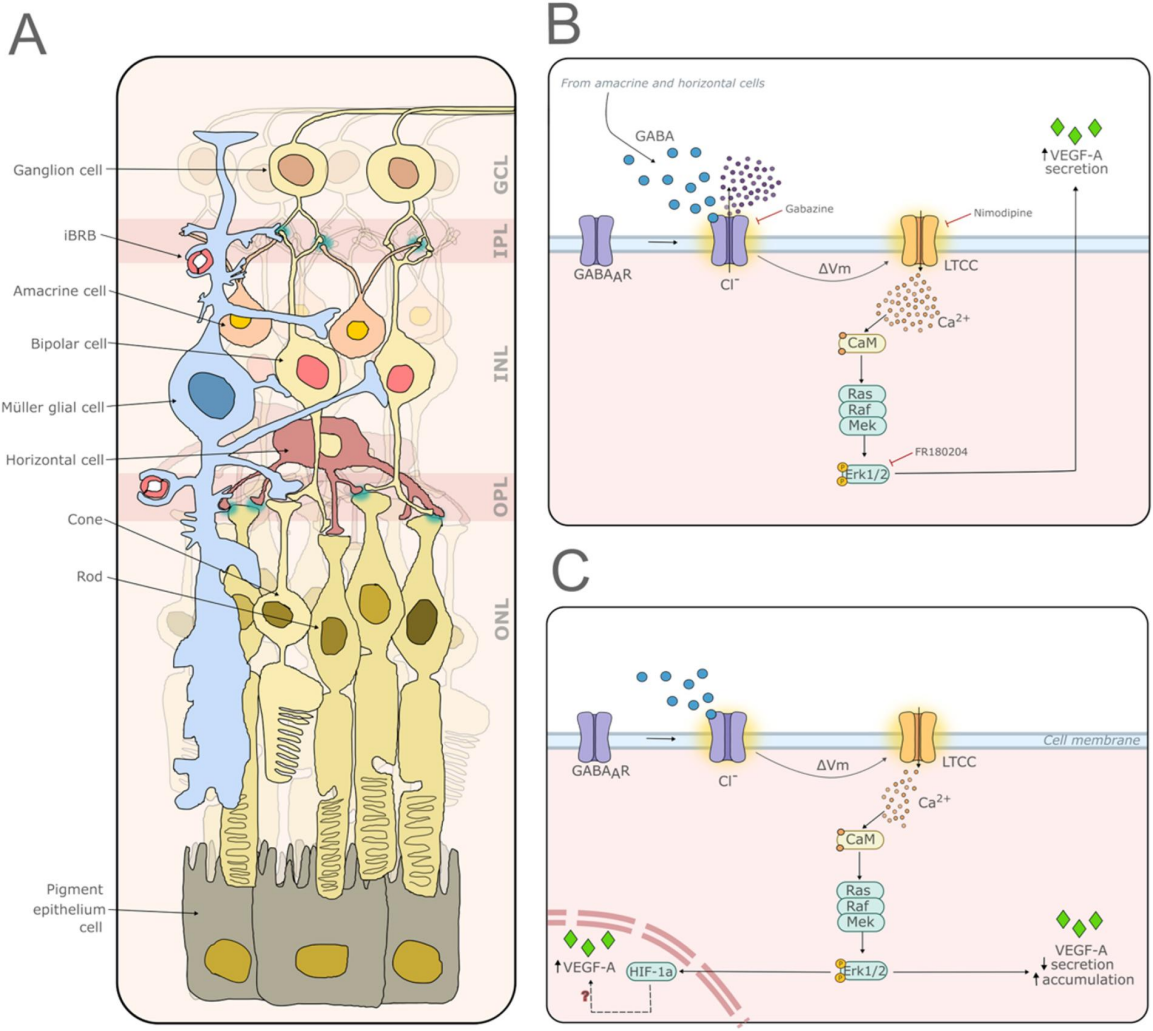
Illustration summarizing the proposed interaction between GABA_A_ receptor activation and VEGF-A dynamics in Müller glia. (A) Schematic drawing of the retina indicating major layers: outer nuclear layer (ONL), outer plexiform layer (OPL), inner nuclear layer (INL), inner plexiform layer (IPL), and ganglion cell layer (GCL). Cyan highlights representative GABAergic synapses (horizontal–photoreceptor and amacrine–bipolar/ganglion contacts). (B) Early time point: synaptically released GABA activates Müller cell GABA_A_ receptors, triggering Ca^2+^ entry and a prompt increase in VEGF-A secretion (e.g., observed at ∼30 min). (C) Late time point (48 h): sustained GABAergic stimulation reduces extracellular VEGF-A while promoting intracellular VEGF-A accumulation, consistent with altered trafficking and/or synthesis. Arrows indicate the direction of signaling. Illustration created in Inkscape.

Pharmacological experiments indicate that the effects of VEGF-A are mainly mediated by ionotropic GABA_A_ receptors. Muscimol, a GABA_A_ agonist, mimicked GABA’s actions on intracellular VEGF-A accumulation and reduced VEGF-A release, while gabazine (a GABA_A_ antagonist) abolished these effects. Conversely, neither the GABA_B_ antagonist CGP55845 nor the GABArho antagonist TPMPA blocked the GABA-induced modulation, suggesting that GABA_B_ or GABArho receptors do not play a major role in these phenomena. Baclofen (a GABA_B_ agonist) did decrease VEGF-A release, indicating that GABA_B_ receptors may modulate secretion under certain conditions, but they do not explain the primary GABA-driven increase in intracellular VEGF-A observed. The GABA concentrations producing maximal effects in our cultures (50–100 µM) are within the range used to evoke GABA_A_ currents in dissociated human Müller cells (Biedermann et al., 2004) and are relevant to disease conditions: vitreous GABA levels are elevated in proliferative diabetic retinopathy (PDR) (Deng et al., 2000), and ischemic models show significant GABA release from inner-retinal neurons (Deng et al., 2000; Kezuka et al., 2003; Neal et al., 1994). Therefore, excess extracellular GABA in disease states could activate MGC GABA_A_ receptors and disrupt VEGF-A homeostasis *in vivo*. Figure 9 illustrates the findings of this study.

Our data indicate that extracellular Ca^2+^ influx is crucial for GABA_A_R-induced regulation of VEGF-A. The effect was absent in Ca^2+^-free Ringer-Krebs solution or when nimodipine was used, indicating that activation of LTCCs downstream of GABA_A_R-mediated depolarization is essential. This process (GABA_A_R activation/membrane depolarization/opening of VGCCs/Ca^2+^ influx) has been observed in other glial cells, such as astrocytes and oligodendrocyte precursor cells, and aligns with reports of LTCC expression and nimodipine sensitivity in Müller cells (Gilbert et al., 1984; Kettenmann et al., 1984; Puro et al., 1996; Young et al., 2010). Our findings thus identify LTCC-dependent Ca^2+^ entry as a key link connecting GABA_A_ activation to downstream secretory functions in MGC.

Blocking ERK1/2 kinase activity with FR180204 significantly reduced the GABA-induced changes in VEGF-A, indicating that ERK1/2 phosphorylation is a key downstream step of the Ca^2+^ signal. These findings align with previous reports showing that Ca^2+^ transients activate MAPK pathways in glial progenitors and glial cells, thereby linking membrane events to transcriptional or post-translational regulatory processes (Pende et al., 1997; Nguyen et al., 2001). In our experiments, ERK1/2 activity appears necessary for the GABA-driven modulation of VEGF-A, although it remains to be determined whether ERK1/2 primarily affects vesicular trafficking, secretory machinery, or VEGF-A processing.

Although GABA_A_ receptors are traditionally inhibitory in mature neurons, in many glial subtypes, including Müller glia, intracellular Cl^-^ accumulation driven by the Na^+^–K^+^–2Cl^-^ cotransporter (NKCC1) makes GABA_A_ opening depolarizing rather than hyperpolarizing (Kettenmann & Schachner, 1985; Plotkin et al., 1997; Young et al., 2010). This depolarization can activate voltage-gated calcium channels, specifically nimodipine-sensitive L-type Ca^2+^ channels described in MGC and related glial preparations, resulting in a rapid influx of extracellular Ca^2+^ (Gadea et al., 2002; Meini et al., 2008; Pérez-García et al., 2004; Schliess et al., 1996; Sticozzi et al., 2013). When intracellular Ca^2+^ levels increase, canonical Ca^2+^/calmodulin-dependent signaling pathways likely lead to MAPK/ERK activation: Ca^2+^ binds calmodulin, activating CaMKK/CaMKI or CaMKII, which can then engage upstream components of the Ras–Raf–MEK–ERK cascade or operate through calmodulin-dependent scaffolds that promote ERK phosphorylation. Together, these steps outline a coherent pathway by which GABA_A_-mediated ionic events in Müller glia translate into nimodipine-sensitive Ca^2+^ signals and subsequent ERK activation.

Hypoxia-inducible factor 1α (HIF-1α) is a key transcriptional regulator that acts as a cellular oxygen sensor and promotes the expression of pro-angiogenic factors like VEGF-A in MGC (Lee et al., 2022; Rodrigues et al., 2013; Xin et al., 2013). Besides its usual stabilization during hypoxia, HIF-1α can also be activated under pseudo-hypoxic metabolic states, such as those linked to the Crabtree effect, where cultured mammalian cells switch from oxidative phosphorylation to aerobic glycolysis (Iommarini et al., 2017; Tan et al., 2024). In this study, control cultures showed minimal nuclear HIF-1α immunoreactivity, indicating no strong baseline Crabtree-type reprogramming. However, a 48-hour exposure to GABA or muscimol led to increased nuclear HIF-1α in Müller glial cells. Therefore, while our data suggest that GABAAR activation and Ca2+ signaling are enough to promote VEGF-A expression in this in vitro system, we cannot rule out that HIF-1α also plays a role, either fully or partially, in the modest transcriptional upregulation of VEGF-A under these conditions. It is worth discussing the metabolic pathways connecting GABA to HIF-1α. Mechanistically, extracellular GABA taken up by MGC may be metabolized through the GABA shunt (GABA-GABA transaminase- succinic semialdehyde-succinate), which feeds succinate into the tricarboxylic acid cycle (Cavalcanti-de-Albuquerque et al., 2022; Tannahill et al., 2013). Succinate buildup is known to inhibit prolyl-hydroxylases (PHDs), stabilizing HIF-1α even in normal oxygen conditions (Atallah et al., 2022; Tannahill et al., 2013). However, the ability of MGC to increase HIF-1α with muscimol treatment suggests that GABA_A_R-evoked Ca^2+^ transients alone are sufficient to enhance HIF-1α synthesis via a Ca^2+^-dependent mechanism. This calcium- and ERK1/2-dependent stabilization of HIF-1α has been observed in both glial and non-glial cells and is associated with HIF-1α-Ser641/643 phosphorylation and mTOR-related pathways (Hui et al., 2006; Koukoulas et al., 2021; Mottet et al., 2003; Mylonis et al., 2006). To determine the relative contributions of these metabolic versus Ca^2+^-dependent routes, further research is needed; for example, siRNA-mediated HIF-1α knockdown, nuclear/cytoplasmic fractionation and western blotting, metabolic tracing or succinate measurement, pharmacological blockade of the GABA shunt, and confirming findings under physiologically relevant O_2_ levels or in retinal explants.

We observed a quick (around 30 min) increase in both intracellular VEGF-A immunoreactivity and secreted VEGF-A after GABA application, with no detectable changes in *Vegfa* mRNA at the same time point. This pattern strongly suggests the mobilization and release of a pre-existing VEGF-A pool through a Ca^2+^- and GABA_A_R-dependent mechanism rather than new transcriptional upregulation. This process is similar to other gliotransmitter and trophic factor release mechanisms in Müller cells (e.g., ATP, D-serine, and glutamate release triggered by purinergic or depolarizing stimuli), which are thought to operate via vesicle-dependent pathways (de Melo Reis et al., 2008; Freitas & de Melo Reis, 2017; Metea & Newman, 2006; Lohr et al., 2014). The rapid time course supports the idea of an immediate gliosecretory response that could have direct physiological or pathophysiological effects.

The cascade we describe (GABA_A_R activation, membrane depolarization, VGCC opening, Ca^2+^ influx, and MAPK signaling) mirrors mechanisms seen in oligodendroglial lineage cells and some secretory endocrine systems like pancreatic β-cells, where ligand-gated depolarization links to Ca^2+^-dependent vesicle mobilization (Gilbert et al., 1984; Kirchhoff & Kettenmann, 1992; Pende et al., 1997). However, to our knowledge, the connection between a neurotransmitter receptor and the controlled release of an angiogenic growth factor has not been reported in retinal glia before. Therefore, our findings broaden the understanding of glial secretory functions and imply that neurotransmitter receptors can directly affect neurovascular signaling.

Since Müller cells are a major source of retinal VEGF-A and are positioned to detect synaptic and vascular microenvironments (Reichenbach & Bringmann, 2013; Saint-Geniez et al., 2008), GABA_A_R-dependent regulation of VEGF-A could have important consequences. Normally, a rapid and localized release of VEGF-A by MGCs in response to neuronal GABAergic activity might support neurovascular coupling or provide short-term trophic effects. However, in disease conditions characterized by elevated extracellular GABA, such as ischemia or diabetic retinopathy, sustained GABAergic activation of MGCs could disturb VEGF-A balance and result in vascular leakage or new vessel formation. Importantly, the buildup of intracellular VEGF-A alongside decreased secretion in certain situations points to a complex regulation of VEGF-A trafficking and release, likely affected by stimulus intensity, receptor subtype activation, or downstream signaling pathways.

Given the close structural coupling where Müller glial processes ensheath GABAergic synapses in both the OPL and IPL (Biedermann et al., 2004), forming a functional tripartite interface, it is reasonable to suggest that synaptic GABA release provides a rate-limiting modulation on glial glutamate clearance. This interaction depends on the unique bioenergetics of Müller cells, which, unlike mature neurons, maintain elevated intracellular chloride levels (∼37 mM) (Bringmann et al., 2013). As a result, GABAergic activation of glial GABA*A* receptors causes chloride efflux and membrane depolarization, a process further amplified by the electrogenic sodium-coupled influx through GABA transporters (GATs) (Eliasof et al., 1998; Malchow et al., 1989; Tempone et al., 2024). Importantly, since the glutamate transporter GLAST functions as a voltage-dependent system that relies on a steep hyperpolarized driving force, this depolarization caused by GABA and the accompanying sodium load oppose glutamate uptake thermodynamically. The outcome is a transient “metabolic–electrical cross-inhibition” that extends the duration of extracellular glutamate, aiding in synaptic integration or spillover (Bergles et al., 2002; Zhang et al., 2007).

Our findings reveal a dynamic “GABA switch” that controls VEGF-A availability, expanding its role beyond angiogenesis to include a vital neuroprotective function within the neurovascular unit. Under normal conditions, the quick release of VEGF-A (∼30 min) probably facilitates neurovascular coupling, where Müller glia detect neuronal GABA activity and rapidly secrete VEGF-A to keep choriocapillaris fenestration intact and meet neuronal metabolic needs (Foxton et al., 2013). In contrast, during diseases marked by sustained GABA increase (such as ischemia or diabetic retinopathy), the shift toward intracellular VEGF-A buildup rather than release (seen at 48 hours) indicates an adaptive response. We suggest this sequestration has a dual purpose: it limits excessive VEGF-A release that could compromise the blood–retinal barrier, while also creating a neuroprotective reserve. This intracellular store helps Müller cells support retinal ganglion cell survival through autocrine and paracrine trophic signals once acute stress exceeds normal levels. Therefore, GABAergic activation of L-type Ca^2+^ channels acts like a rheostat, adjusting VEGF-A balance to promote neuronal survival and reduce vascular permeability during metabolic stress (Figure 9).

This work was conducted in primary mouse Müller cell cultures; therefore, *in vivo* confirmation is necessary to establish physiological relevance and tissue-level effects. Future studies should 1) identify the specific GABA_A_R subunit composition in MGCs, 2) elucidate the molecular mechanisms linking ERK1/2 activation to VEGF-A trafficking and secretion, 3) determine whether the pathway functions in the intact retina and during retinal disease models, and 4) assess whether pharmacological modulation of GABA_A_R signaling in Müller cells can be used therapeutically to reduce pathological angiogenesis without disrupting retinal homeostasis.

In summary, our data reveal a previously unknown mechanism by which GABAergic signaling controls the Ca^2+^–ERK1/2–VEGF-A axis in MGC. These findings suggest that GABA_A_R in Müller cells are involved not only in maintaining ionic balance but also in integrating neurovascular signals, positioning Müller glia as a central hub that connects neurotransmission and retinal angiogenesis. Understanding the *in vivo* role of this pathway and its potential for therapy in vasoproliferative retinopathies is an important area for future research.
